# Clinical performance of LOCATOR® attachments: A retrospective study with 1–8 years of follow‐up

**DOI:** 10.1002/cre2.122

**Published:** 2018-07-27

**Authors:** Christophe Guédat, Ursina Nagy, Martin Schimmel, Frauke Müller, Murali Srinivasan

**Affiliations:** ^1^ Division of Gerodontology and Removable Prosthodontics, University Clinics of Dental Medicine University of Geneva Switzerland; ^2^ Division of Orthodontics University Clinics of Dental Medicine, University of Geneva Switzerland; ^3^ Division of Gerodontology, School of Dental Medicine University of Bern Switzerland; ^4^ Service of Geriatrics, Department of Internal Medicine, Rehabilitation and Geriatrics University Hospitals of Geneva Switzerland

**Keywords:** attachment wear, implant overdentures, LOCATOR® attachments, mechanical complications, overdenture, removable dental prostheses

## Abstract

The use of LOCATOR® attachments in implant‐supported removable dental prostheses (ISRDPs) has been evidenced with conflicting clinical behavior in literature. This retrospective study aimed to investigate the long‐term clinical performance of LOCATOR® attachments by evaluating the frequency of the encountered mechanical complication events (MCEs) and the factors that play a role in attachment wear (AW). The study recruited participants with ISRDPs on LOCATOR® attachments. Clinical parameters, number of MCEs (attachment replacements, attachment loosenings, denture cap‐related events, loss of retention and/or insert, and implant fractures), and AW were recorded. Nonparametric tests were applied for statistical analyses (𝛼=0.05). Baseline demographics for the recruited 47 participants (mean age: 72.0 ± 9.0 years) revealed an implant survival rate of 94.9% (mean observation period: 54.8 months), average peri‐implant probing depths, bleeding on probing scores, and plaque scores of 1.80 ± 1.50 mm, 0.70 ± 0.90, and 0.81 ± 0.90, respectively.

MCEs were directly influenced by the time in use (*p* < 0.001). The most frequently encountered MCEs were loss of retention (*p* < 0.001) and denture cap‐related complications (*p* = 0.004). AW was found to be significantly higher in the maxilla than in the mandible (*p* = 0.028); in the maxilla, the vestibular (*p* = 0.005) and mesial (*p* = 0.01) aspects were the most common wear sites. Maxillary implant overdentures revealed more vestibular AW (*p* = 0.013). In prostheses supported by >3 implants, vestibular (*p* = 0.046) and mesial (*p* = 0.032) AW were common. Lingual AW (*p* = 0.021) was observed more frequently when the support was <3 implants. Loss of retention and AW are the most common complications encountered with LOCATOR® attachments. Therefore, a modification in the attachment design along with an amelioration of the attachment surface may help decrease the maintenance needs and further enhance its clinical performance.

## 1 INTRODUCTION

Implant‐supported removable dental prostheses (ISRDPs) therapy for the restoration of the edentulous jaws is a well‐documented therapy with high rates of treatment success and patient satisfaction (Emami, Heydecke, Rompre, de Grandmont, & Feine, 2009). Implant overdentures (IODs) have proven to be significantly useful especially in the elderly population demonstrating high patient satisfaction, comfort, prostheses stability, and chewing function (Awad et al., 2003; van Kampen, van der Bilt, Cune, Fontijn‐Tekamp, & Bosman, 2004; Visser, Raghoebar, Meijer, Batenburg, & Vissink, 2005). Success of ISRDPs is not just limited to completely edentulous patients but also extends to the domain of partially edentulous jaws (El Mekawy, El‐Negoly, Grawish Mel, & El‐Hawary, 2012; Jensen, Meijer, Raghoebar, Kerdijk, & Cune, 2017).

The success of ISRDPs, however, depends on a multitude of factors and the treatment planning needs to be adapted to suit the patient's age, functional state, general health, socioeconomic context, and perhaps the attachment system itself. The LOCATOR® system has been reported to be the most frequently used stud‐type attachment system for IODs by an international survey (Kronstrom & Carlsson, 2017). This system has been evidenced with good clinical performance (Cakarer, Can, Yaltirik, & Keskin, 2011; Kappel, Giannakopoulos, Eberhard, Rammelsberg, & Eiffler, 2016; Mackie, Lyons, Thomson, & Payne, 2011; Wang et al., 2016; Zou et al., 2013), along with patient satisfaction and improved quality of life (Fernandez‐Estevan, Montero, Selva Otaolaurruchi, & Sola Ruiz, 2017). However, excessive wear, loss of retention, increased maintenance requirements, decline in the retentive capacities in cases with nonparallel implant divergences, and diverse conflicting clinical behavior have been frequently reported (Abi Nader et al., 2011; Al‐Ghafli, Michalakis, Hirayama, & Kang, 2009; Alsabeeha, Swain, & Payne, 2011; Cune, van Kampen, van der Bilt, & Bosman, 2005; Evtimovska, Masri, Driscoll, & Romberg, 2009; Kleis, Kammerer, Hartmann, Al‐Nawas, & Wagner, 2010; Kobayashi et al., 2014; Rutkunas, Mizutani, & Takahashi, 2005; Rutkunas, Mizutani, Takahashi, & Iwasaki, 2011; Srinivasan et al., 2016; Visser et al., 2005).

Hence, the primary aim of this retrospective study was to investigate the reasons relating to the increased maintenance requirements of the LOCATOR® attachments by assessing the frequency of the mechanical complications encountered and its influencing factors. A secondary and a more specific aim of this study was to identify the factors that may play a role in the wear of this attachment. Therefore, the primary null hypothesis set for this retrospective study was that the number of events of mechanical complications encountered with the LOCATOR® attachment is not influenced by the type of the removable prosthesis, the jaw rehabilitated, number of implants supporting the prosthesis, time in use, attachment height, axial inclination of the implants, chewing efficiency, and wear of the attachment. The secondary hypothesis set was that the wear of the LOCATOR® attachment is not influenced by the type of prostheses, the jaw rehabilitated, number of implants supporting the prostheses, time in use, attachment height, axial inclination of the implants, and chewing efficiency.

## 2 METHODS

The study protocol and methodology were independently reviewed and approved by the ethical committee of the University Hospitals of Geneva, Switzerland (CER no. 14‐046). Furthermore, the study was conducted with strict adherence to all ethical principles and has been reported in compliance to the STROBE (Strengthening the Reporting of Observational Studies in Epidemiology) standards/checklists (von Elm et al., 2008).

### 2.1 Study design

The present study was designed as a retrospective, single center, clinical study on human subjects.

### 2.2 Setting

The study was conducted in the University Clinics of Dental Medicine, University of Geneva, Switzerland, between May and November 2014. Patients included during this period had their implants loaded and their prostheses in situ for a minimum of 1 year.

### 2.3 Participants

Participants included in this retrospective study were from an existing pool of patients treated in the student clinics of Division of Gerodontology and Removable Prosthodontics at the University Clinics Dental of Medicine, University of Geneva, Switzerland. They were included if they satisfied the following inclusion criteria:

- Patient must be total or partially edentulous in either or both jaws.
- Patient must be rehabilitated with an ISRDP engaging LOCATOR® attachment/s.
- The ISRDP must have been loaded and in function for a minimum of one year.

Participants were excluded if they presented with

- unwillingness to sign the consent form.
- debilitating health problems.
- severe cognitive impairment.
- uncontrolled diabetes.
- history of orofacial neoplasia.

Post hoc exclusion criteria:

- not original manufacturer parts (third‐party components).
- group sample size too small (<5 cases).

#### 2.3.1 Participants groups

The included participants were allocated into one of the four study groups according to their type of ISRDPs in situ:

1. Group 1 (Ci/)—Maxillary implant‐supported complete removable dental prostheses group.
2. Group 2 (/Ci)—Mandibular implant‐supported complete removable dental prostheses group.
3. Group 3 (Pi/or/Pi)—Maxillary or Mandibular implant‐supported removable partial dental prostheses group.
4. Group 4 (Ci/Ci)—Maxillary and mandibular implant‐supported complete removable dental prostheses group.

### 2.4 Variables

#### 2.4.1 Primary endpoint/measure(s)

The primary outcome measure for this retrospective study was the mechanical complication rate encountered with the LOCATOR® attachments and its influencing factors. Mechanical complications were defined for the purpose of this study as complications, occurring in the prefabricated components of the attachments as well as the implants, caused by mechanical forces. This has been adapted from the definition of mechanical risks (Salvi & Bragger, 2009). Hence, all mechanical complication events (MCEs) occurring with the attachment, denture cap, retention insert, and implant were recorded and classified accordingly. These hardware complications were evaluated under various influencing factors:

1. Type of ISRDPs used in rehabilitation: the ISRDPs type included, implant‐supported complete removable dental prosthesis (upper or lower) and partial removable dental prostheses (upper or lower).
2. Jaw rehabilitated: in the maxilla or mandible.
3. Number of implants supporting the prostheses.
4. Time in use: total time (in months), the prostheses has been in situ, in function since loading.
5. Attachment height: height of the gingival cuff of the LOCATOR® attachment.
6. Axial inclination of the implants: The axial inclination of the implants, for the purpose of this study, has been classified either as parallel or inclined. For convenience, any angular discrepancy between zero degrees up to a maximum of 10 degrees was considered as parallel, while angular discrepancies exceeding 10 degrees up to a maximum of 20 degrees were considered as inclined for the purpose of this study.
7. Wear of the attachment under four categories:
  - No wear—no visible wear.
  - Minimal wear—visible scratches on the attachment surface but limited to the surface coating.
  - Moderate wear—more pronounced wear, resulting in loss of the surface coating and exposing the bare metallic surface underneath.
  - Advanced—severe wear, resulting in damage to the shape of the attachment.

Wear was further site‐specifically classified as vestibular, mesial, distal, and lingual wear.

#### 2.4.2 Secondary endpoint/measure(s)

The secondary and a more specific measure was the associated attachment wear and the influencing factors including type of prosthesis, jaw rehabilitated, number of implants, time in use, attachment height, axial inclination, and chewing efficiency.

#### 2.4.3 Tertiary endpoint/measure(s)

The tertiary measures included implant survival, peri‐implant bleeding on probing scores, peri‐implant plaque scores, and peri‐implant probing depths. Implant survival/success criteria adopted for this study were as described by Buser, Weber, and Lang (1990), as the absence of mobility, pain, recurring peri‐implant infection, and continued radiolucency around implant. The time of failure of the implant was classified as early, delayed, or late (ten Bruggenkate, Asikainen, Foitzik, Krekeler, & Sutter, 1998). Peri‐implant bleeding on probing scores and plaque scores were assessed using the modified plaque and bleeding index (Mombelli, van Oosten, Schurch Jr., & Land, 1987).

### 2.5 Data sources/measurements

A list of prospective participants was generated from the existing clinic database, and patients were first contacted by a conventional mail and later on followed up by a telephone call. The willing participants were invited for a screening visit and were included if they satisfied the study inclusion criteria. A signed informed consent was then obtained from the included patients, who were then allotted into one of the earlier mentioned participant groups. At the clinical appointment, all participants underwent a brief intraoral dental examination and further specific examinations related to each of the outcome measures described. All examinations were performed by a team of two investigators (C. G. and U. N.). Further, patient dental records were checked and all past mechanical events, implant events, were recorded in the clinical record forms. All the data collected were tabulated in electronic spreadsheet electronically (MS‐Excel 2016 for Macintosh, version 16.0, Microsoft Corporation, Redmond, WA, USA).

### 2.6 Study size

Fifty participants consented to participate and were available for clinical examination.

### 2.7 Quantitative variables

Quantitative variables in the study included age, chewing efficiency, plaque and bleeding scores, probing depth, number of implants, time in use, and time until events. In each subanalysis and in tables where these variables were included, their handling was described there, including if any of those variables was categorized or transformed.

### 2.8 Statistical methods

The collected data were verified for a normal distribution, and appropriate nonparametric tests were applied for significance between the study parameters. The level of confidence was set to 95%. No sensitivity analyses were applied for this retrospective study.

Two groups of outcome variables were retained: types of wear and mechanical complications. For wear, implants had their vestibular, mesial, distal, and lingual wear recorded. For attachment height and attachment inclination, the frequency of implants with each type of wear was compared between the levels of each covariate at the implant‐level. For patient characteristics (such as the chewing efficiency, the number of implants per patient, the “jaw group,” prosthesis type, and years in use), the number of patients with wear (of each type) was compared between levels of those independent variables.

Six types of mechanical complications were recorded during the study: attachment replacement, attachment loosening, denture cap‐related problem, loss of retention, loss of insert, and implant fracture. Associations between the occurrence of these mechanical complications and patient characteristics were assessed. Thus, the frequency of patients experiencing each event was compared across levels of each patient characteristic, such as prosthesis group or the jaw rehabilitated.

For our analyses, comparisons of frequencies of patients or implants between levels of categorical variables were assessed using Fisher's exact test, whereas the distribution of continuous variables across those groups was compared using Kruskal–Wallis' test.

Our analyses did not include interactions, and the same subset of patients was used for all the analyses.

Cases of missing data were minimal and were addressed in each table or relevant subsection. Given the study goals, simple analytical approaches were favored. There was no focus on causality, and there were no major choices in the statistical methodology that warranted sensitivity analyses.

## 3 RESULTS

### 3.1 Participants

**Figure 1:**
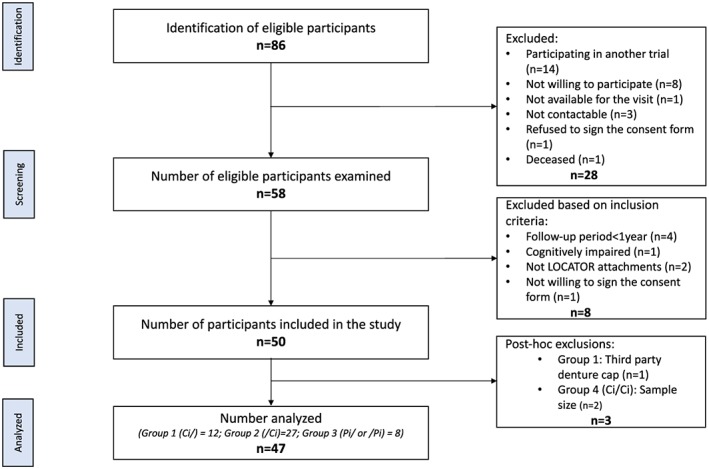
Flow diagram of the entire participant identification, screening, and inclusion process (n: number; Ci/: maxillary implant‐supported complete removable dental prostheses [ISCRDPs]; /Ci: mandibular ISCRDPs; Ci/Ci: maxillary and mandibular ISCRDPs; Pi/: maxillary implant‐supported removable partial dental prostheses (ISRPDPs); /Pi: mandibular ISRPDPs)

A total of 86 potentially eligible participants were contacted for participation in the study, 58 subjects were screened, and 50 willing participants who satisfied the inclusion criteria were included in the study and were allocated into one of the four study groups. The entire participant identification, screening, and inclusion process, along with reasons for exclusion, is shown in a flow diagram (Figure 1). The participant information collected are listed in Table 1.

**Table 1:** Baseline demographics of the study cohort

Groups	Number of participants			Age range (Mean ± *SD*)	Number of implants placed (P), failed (F), survived (S), implant survival rate percentage (SR%), and mean (x) number of implants per participant					Time of implant failure			Follow‐up period in months (Mean ± *SD*)	Implant plaque score (Mean ± *SD*)	Implant bleeding score (Mean ± *SD*)	Implant probing depth (Mean ± *SD*)
	M	W	T		P	F	S	SR%	$\bar{x}$	E	D	L				
Ci/‐	6	6	12	67.8 ± 8.6	50	4	46	92.0	4.2	2	1	1	58.2 ± 22.8	0.62 ± 0.80	0.40 ± 0.70	2.40 ± 1.70
‐/Ci	14	13	27	74.4 ± 9.8	56	1	55	98.2	2.1	1	0	0	54.8 ± 22.8	1.07 ± 0.87	0.89 ± 1.00	1.40 ± 1.10
Pi/‐ or ‐/Pi	1	7	8	70.4 ± 8.1	12	1	11	91.7	1.5	1	0	0	49.5 ± 19.8	0.64 ± 0.83	0.66 ± 0.75	1.36 ± 1.50
Total	21	26	47	72.0 ± 9.5	118^a^	6	112	94.9	2.5	4	1	1	54.8 ± 22.1	0.81 ± 0.90	0.70 ± 0.90	1.80 ± 1.50
*p* value	0.131^b^			0.191^c^								0.479^c^		**0.007** c	**0.036** c	**0.006** c

*Note*. M: men; W: women; T: total; *SD*: standard deviation; SR%: survival rate percentage; E: early failure; D: delayed; L: late failures Ci/‐: maxillary implant‐supported complete removable dental prostheses (CRDPs); ‐/Ci: mandibular implant‐supported CRDPs; Pi/‐: maxillary implant‐supported removable partial dental prostheses (ISRPDPs); ‐/Pi: mandibular ISRPDPs.

a Includes 4 implants that were replaced after implant failures.

b Fisher's test.

c Kruskal–Wallis test.

### 3.2 Descriptive and outcome data

From the total of 50 included participants, three participants were excluded from the study (post hoc exclusion). Two of these excluded participants were from Group 4 (*n* = 2) because the sample size was too small for any meaningful comparison, and one participant from Group 1 with six implants because the denture caps fixed in her prosthesis were not from the original manufacturer. Therefore, a total of 47 participants were finally included and analyzed (*n* = 47; ♂ = 21, ♀ = 26; mean age ± *SD* = 72.0 ± 9.5); the participants' baseline demographics are given in Table 1.

### 3.3 Mechanical complications

Overall MCEs when analyzed for time in use demonstrated a significant relationship (*p* < 0.001; Fisher's test, Table 2). The specific mechanical complications associated with the loss of retention (insert changes) and denture cap problems were also significant with time in use (Table 2).

**Table 2:** Mechanical complication events (MCEs) corrected for the time in use

Time in use (years)	Number of participants surviving without any mechanical complication event (S), number of mechanical complication events (N), and number of patients (P) in subgroup experiencing at least one of those events													
	Attachment replacement		Attachment loosening		Denture cap‐related event		Loss of retention		Loss of insert		Implant fracture		Any event	
	S	N (P)	S	N (P)	S	N (P)	S	N (P)	S	N (P)	S	N (P)	S	N (P)
0–1	46	1 (1)	47	0 (0)	43	6 (4)	31	19 (16)	47	0 (0)	47	0 (0)	28	26 (19)
1–2	46	0 (0)	46	1 (1)	43	0 (0)	25	10 (10)	46	1 (1)	47	0 (0)	22	12 (10)
2–3	46	0 (0)	46	0 (0)	43	0 (0)	20	16 (14)	46	0 (0)	47	0 (0)	17	16 (14)
3–4	46	0 (0)	46	0 (0)	43	0 (0)	19	9 (8)	46	0 (0)	47	0 (0)	16	9 (8)
4–5	45	1 (1)	46	0 (0)	41	2 (2)	15	13 (10)	46	0 (0)	47	0 (0)	13	16 (10)
5–6	45	0 (0)	46	0 (0)	41	0 (0)	15	5 (4)	46	0 (0)	47	0 (0)	13	5 (4)
6–7	44	1 (1)	46	0 (0)	41	0 (0)	15	3 (3)	46	0 (0)	46	1 (1)	13	5 (3)
7–8	44	0 (0)	46	0 (0)	41	0 (0)	15	0 (0)	46	0 (0)	46	0 (0)	13	0 (0)
Total		3 (3)		1 (1)		8 (6)		75 (32)		1 (1)		1 (1)		89 (34)
Years until first event, (Mean ± *SD*)		3.5 ± 2.9		1.9 (NA)		1.7 ± 2.0		1.6 ± 1.4		1.8 (NA)		6.7 (NA)		1.4 ± 1.3
Events per patient, (Mean ± *SD*)		0.1 ± 0.2		0.0 ± 0.1		0.1 ± 0.5		1.6 ± 1.6		0.0 ± 0.1		0.0 ± 0.1		1.9 ± 1.9
*p* value		1^a^		1^a^		**0.004** a		**<0.001** a		1		1		**<0.001** a

*Note*. The *p* value corresponds to a Fisher's test, with the null hypothesis being that the rate of patients experiencing each type of mechanical complication is equal regardless to the number of years in function: standard deviation.

a Fisher's test.

There were no significant differences for overall MCEs when analyzed for the type of ISRDPs (*p* = 1.000; Fisher's test), jaw rehabilitated (*p* = 0.503; Fisher's test), number of implants (*p* = 0.358; Fisher's test), axial inclination of implants (*p* = 1.000; Fisher's test), and chewing efficiency (*p* = 0.748; Fisher's test [Tables A1, A2, A3, A4, A5]).

MCEs corrected for wear were not significant (*p* = 0.187; Fisher's test; Table A6), but a trend can be interpreted from the results. In patients with no observed wear on the attachments, 52.4% were associated with retention loss, whereas all patients (100%) with advanced wear presented with a loss of retention.

### 3.4 Attachment wear

When corrected for the type of prostheses, the overall AW demonstrated no difference between the groups (Table 3). However, significantly higher rates of vestibular wear (83.3%) was observed in Group 1 as opposed to the other groups (*p* = 0.013, Fisher's test). Although the highest rates of mesial wear (66.7%) was also seen in Group 1, it was not statistically significant.

**Table 3:** Attachment wear corrected for prostheses type, jaw rehabilitated, and the number of implants per prostheses

	Groups	Number of participants (*n*)	Number of sites (implants survived; *n*)	Follow‐up period in months (Mean ± *SD*)	Number of patients (P) and share of patients (%) showing wear across the attachment split into four locations and overall (for any type of wear) per attachment. Mean wear ( $\bar{x}$) ± *SD*								
					Vestibular		Mesial		Distal		Lingual		Overall
					P (%)	$\bar{x}\;$ ± *SD*	P (%)	$\bar{x}\;$ ± *SD*	P (%)	$\bar{x}\;$ ± *SD*	P (%)	$\bar{x}\;$ ± *SD*	P (%)
Type of prosthesis groups	Ci/‐	12	45	58.2 ± 22.8	10 (83.3)	0.97 ± 0.71	8 (66.7)	0.54 ± 0.57	4 (33.3)	0.33 ± 0.50	2 (16.7)	0.10 ± 0.23	7 (58.3)
	‐/Ci	27	55	54.8 ± 22.8	9 (33.3)	0.44 ± 0.75	8 (29.6)	0.30 ± 0.52	6 (22.2)	0.30 ± 0.64	4 (14.8)	0.30 ± 0.76	8 (29.6)
	Pi/‐ or ‐/Pi	8	11	49.5 ± 19.8	4 (50.0)	0.88 ± 0.99	2 (25.0)	0.50 ± 0.93	4 (50.0)	1.13 ± 1.25	4 (50.0)	1.13 ± 1.25	4 (50.0)
	Total	47	111	54.8 ± 22.1	23 (48.9)	0.65 ± 0.81	18 (38.3)	0.39 ± 0.61	14 (29.8)	0.45 ± 0.79	10 (21.3)	0.39 ± 0.84	26 (55.3)
	*p* value			0.479^b^	**0.013** a		0.08^a^		0.302^a^		0.146^a^		0.082^b^
Type of jaw rehabilitated	Maxilla	15	48	56.5 ± 22.2	12 (80.0)	1.04 ± 0.78	10 (66.7)	0.70 ± 0.74	6 (40.0)	0.53 ± 0.75	4 (26.7)	0.34 ± 0.70	9 (60.0)
	Mandible	32	63	54.0 ± 22.3	11 (34.4)	0.47 ± 0.76	8 (25.0)	0.25 ± 0.49	8 (25.0)	0.41 ± 0.82	6 (18.8)	0.41 ± 0.90	10 (31.3)
	Total	47	111	54.8 ± 22.1	23 (48.9)	0.65 ± 0.81	18 (38.3)	0.39 ± 0.61	14 (29.8)	0.45 ± 0.79	10 (21.3)	0.39 ± 0.84	26 (55.3)
	*p* value			0.545^b^	**0.005** a		**0.01** a		0.324^a^		0.704^a^		**0.028** a
Number of implant/s per prosthesis (*n*)	1	6		51.0 ± 16.0	3 (50.0)	1.00 ± 1.10	2 (33.3)	0.67 ± 1.03	3 (50.0)	1.00 ± 1.10	3 (50.0)	1.00 ± 1.1	3 (50.0)
	2	30		55.0 ± 22.8	11 (36.7)	0.47 ± 0.73	8 (26.7)	0.27 ± 0.50	7 (23.3)	0.37 ± 0.79	5 (16.7)	0.37 ± 0.88	10 (33.3)
	3	1		44.7 ± NA	0 (0.0)	0.00 ± NA	0 (0.0)	0.00 ± NA	0 (0.0)	0.00 ± NA	0 (0.0)	0.00 ± NA	0 (0.0)
	4	7		50.9 ± 27.1	6 (85.7)	1.00 ± 0.72	5 (71.4)	0.58 ± 0.55	2 (28.6)	0.33 ± 0.58	0 (0.0)	0.00 ± 0.00	4 (57.1)
	5	1		61.3 ± NA	1 (100.0)	2.00 ± NA	1 (100.0)	1.60 ± NA	1 (100.0)	0.80 ± NA	0 (0.0)	0.00 ± NA	1 (100)
	6	2		78.8 ± 7.5	2 (100.0)	0.79 ± 0.65	2 (100.0)	0.42 ± 0.12	1 (50.0)	0.42 ± 0.59	2 (100.0)	0.58 ± 0.12	1 (50.0)
	Total	47		54.8 ± 22.1	23 (48.9)	0.65 ± 0.81	18 (38.3)	0.39 ± 0.61	14 (29.8)	0.45 ± 0.79	10 (21.3)	0.39 ± 0.84	26 (55.3)
	*p* value			0.478^b^	**0.046** a		**0.032** a		0.366^a^		**0.021** a		0.167^a^

The *p* value corresponds to a Fisher's test comparing the frequency of patients with wear among the different categories of patients, with the null hypothesis being that the share of patients with wear is equal across groups. The Kruskal–Wallis test compared the distribution of the follow‐up period between the groups. Ci/: upper implant‐supported CRDP; /Ci: lower implant‐supported CRDP; Pi/: upper implant‐supported PRDP; /Pi: lower PRDP *p* test; *n*: number; *SD*: standard deviation.

a Fisher's test.

b Kruskal–Wallis test.

Overall wear of the attachments was found to be significantly higher for the maxilla as opposed to the mandible (*p* = 0.028, Fischer's test). The vestibular wear was found to be significantly higher for the maxilla than the mandible (*p* = 0.005, Fisher's test), as well as for the mesial wear (*p* = 0.010, Fisher's test, Table 3). There were no differences between the jaws for lingual and distal wear of the attachments.

A higher number of implants were associated with a tendency for a higher prevalence of vestibular wear (*p* = 0.046, Fisher's test) and statistically significant mesial wear (*p* = 0.032, Fisher's test; Table 3). A reverse phenomenon was observed for lingual wear, that is, lower number of implants exhibited higher wear (*p* = 0.021, Fisher's test; Table 3).

The percentage of implants with wear, for all sites (vestibular, mesial, distal, and lingual), tends to increase with time in use. The percentage of patients with wear on their attachments remain below 40% up until the first 5 years of use. Thereafter, it increases; by the 7th and 8th year of use, almost all patients demonstrate wear in all sites. The overall total attachment wear was found to be significant with time in use (*p* = 0.001, Fisher's test). The site‐specific wear as opposed to time in use was significant for vestibular (*p* = 0.013), mesial (*p* = 0.011), and distal (*p* = 0.023) sites. Lingual sites only demonstrated a tendency (*p* = 0.049, Fisher's test; Table 4).

**Table 4:** Attachment wear corrected for the time in use and implant inclination

		Number of participants (*n*)	Number of sites (implants survived) (*n*)	Number of patients (P) and share of patients (%) showing wear across the attachment split into four locations and overall (for any type of wear) per attachment. Mean wear ( $\bar{x}$) ± *SD*								
				Vestibular		Mesial		Distal		Lingual		Overall
				P (%)	$\bar{x}\;$ ± *SD*	P (%)	$\bar{x}\;$ ± *SD*	P (%)	$\bar{x}\;$ ± *SD*	P (%)	$\bar{x}\;$ ± *SD*	P (%)
Time in use per patient (years)	1–2	6	15	1 (16.7)	0.08 ± 0.20	1 (16.7)	0.17 ± 0.41	0 (0.0)	0.00 ± 0.00	0 (0.0)	0.00 ± 0.00	0 (0.0)
	2–3	3	6	0 (0.0)	0.00 ± 0.00	0 (0.0)	0.00 ± 0.00	0 (0.0)	0.00 ± 0.00	0 (0.0)	0.00 ± 0.00	0 (0.0)
	3–4	6	12	2 (33.3)	0.42 ± 0.80	0 (0.0)	0.00 ± 0.00	1 (16.7)	0.33 ± 0.82	1 (16.7)	0.33 ± 0.82	1 (16.7)
	4–5	8	16	3 (37.5)	0.56 ± 0.82	2 (25.0)	0.31 ± 0.70	1 (12.5)	0.25 ± 0.71	1 (12.5)	0.25 ± 0.71	3 (37.5)
	5–6	15	37	9 (60.0)	0.87 ± 0.86	9 (60.0)	0.69 ± 0.71	6 (40.0)	0.42 ± 0.61	3 (20.0)	0.37 ± 0.77	9 (60.0)
	6–7	5	13	5 (100.0)	1.15 ± 0.55	3 (60.0)	0.47 ± 0.62	3 (60.0)	1.17 ± 1.25	2 (40.0)	0.70 ± 1.30	4 (80.0)
	7–8	3	10	3 (100.0)	1.44 ± 1.08	3 (100.0)	0.78 ± 0.63	3 (100.0)	1.61 ± 0.84	3 (100.0)	1.72 ± 1.18	2 (66.7)
	8–9	1	2	0 (0.0)	0.00 ± 0.00	0 (0.0)	0.00 ± 0.00	0 (0.0)	0.00 ± 0.00	0 (0.0)	0.00 ± 0.00	0 (0.0)
	Total	47	111	23 (48.9)	0.65 ± 0.81	18 (38.3)	0.39 ± 0.61	14 (29.8)	0.45 ± 0.79	10 (21.3)	0.39 ± 0.84	26 (55.3)
	*p* value			**0.013** a		**0.011** a		**0.023** a		0.049^a^		**0.001** a

		Number of participants (*n*)	Number of sites (implants survived; *n*)	Number of sites (S) and share of sites (%) showing wear across the attachment split into four locations and overall (for any type of wear) per attachment. Mean wear ( $\bar{x}$) ± *SD*								
				Vestibular		Mesial		Distal		Lingual		Overall
				S (%)	$\bar{x}\;$ ± *SD*	S (%)	$\bar{x}\;$ ± *SD*	S (%)	$\bar{x}\;$ ± *SD*	S (%)	$\bar{x}\;$ ± *SD*	S (%)
Implant axial inclination per site (degrees)	0–10	42	86	36 (41.9)	0.78 ± 1.00	28 (32.6)	0.78 ± 1.00	18 (20.9)	0.78 ± 1.00	12 (14.0)	0.78 ± 1.00	47 (54.7)
	10–20	15	25	7 (28.0)	0.44 ± 0.77	2 (8.0)	0.44 ± 0.77	4 (16.0)	0.44 ± 0.77	4 (16.0)	0.44 ± 0.77	9 (36.0)
	Total	57	111	43 (38.7)	0.70 ± 0.96	30 (27.0)	0.43 ± 0.76	22 (19.8)	0.41 ± 0.86	16 (14.4)	0.31 ± 0.80	56 (50.5)
	Number of worn implants per patient (Mean ± *SD*)			0.9 ± 1.2		0.6 ± 1.0		0.5 ± 0.8		0.3 ± 0.7		1.2 ± 1.3
	*p* value			0.249^a^		0.020^a^		0.777^a^		0.755^a^		0.116^a^

The *p* value corresponds to a Fisher's test comparing the frequency of patients/sites with wear among the different categories of patients/type of implant (according to site inclination) respectively, with the null hypothesis being that the share of patients/sites with wear is equal across groups. *SD*: standard deviation; *n*: number.

a Fisher's test.

Wear was directly related to attachment height (*p* = 0.041, Fisher's test). Higher attachment leads to a higher overall wear profile. Although no one particular wear site could be identified for a higher tendency, it was observed that vestibular wear demonstrated a higher yet not significant tendency (*p* = 0.050, Fisher's test) for greater wear as compared with the other regions (Tables A7 and A8).

Implants with an axial inclination between 0 and 10 degrees presented 32.6% of mesial wear compared with 8% of wear in implants with higher axe divergences (10–20 degrees; *p* = 0.020, Fisher's test; Table 4). There was no association between axial inclination and other regions of wear (vestibular, distal, and lingual sites).

No significant relationship between attachment wear and chewing efficiency was found (*p* = 0.636, Fisher's test; Table A8).

### 3.5 Implant survival

A total of 118 implants had been placed in 47 patients, where 112 implants survived resulting in an implant survival rate percentage (SR%) of 94.9% in a mean observation period of 54.8 ± 22.1 months. The group‐wise calculated SR% was 92.0%, 98.2%, and 91.7% for Groups 1, 2, and 3 (Table 1).

One patient in Group 1 had two implants failing. They were not replaced because the remaining four implants were considered adequate to support the maxillary IOD. Two patients in Group 1 had one failure each; these implants had been replaced. Two patients (one in Group 2 and one in Group 3) had one implant failure each, and these were also replaced. The calculated implant failure rate between the groups was not statistically significant (*p* = 0.233; Fisher's test). There were four early failures (*n* = 4: Group 1 = 2; Group 2 = 1; Group 3 = 1). There was one delayed and one late failure; both of which were in Group 1.

### 3.6 Plaque scores, bleeding scores, and peri‐implant probing depths

When comparing clinical parameters, there was a significant difference between the groups for the overall peri‐implant plaques scores (*p* = 0.007; Kruskal–Wallis test), bleeding scores (*p* = 0.036; Kruskal–Wallis test), and probing depths (*p* = 0.006; Kruskal–Wallis test) as shown in Table 1.

## 4 DISCUSSION

### 4.1 Critique of the methods

As in any retrospective study, there are inherent shortcomings in the study design, which have to be taken into consideration when interpreting the results from this study. Furthermore, the chosen cohort for this survey was heterogeneous, in terms of age, gender, number of natural teeth, and type of implant‐denture provided. Last but not least, different numbers of implants were placed in the study participants and only the implant with the most pronounced wear was used for the statistical analyses. The results are therefore displaced “overcritical,” as in the real clinical situation all second, third, or more implants presented with less wear than described in the results from this paper.

### 4.2 More mechanical complications with time in use

Mechanical complications are bound to increase with time in function, as with all technical equipment. The loss of retention over time has also been confirmed in previous in vitro studies (Kobayashi et al., 2014; Srinivasan et al., 2016). Repeated insertion and removal alter the contacting surfaces, and this leads to loss of the surface material (Kleis et al., 2010), and in turn contributes to the retention loss. This study demonstrated that the MCEs happened mostly in the first years, as in other attachment systems, where adjustments were most frequently encountered in the first year (Cehreli, Karasoy, Kokat, Akca, & Eckert, 2010).

### 4.3 Loss of retention most frequent MCE and denture cap‐related events

The male inserts of the LOCATOR® attachment showed the highest maintenance need, as demonstrated in previous studies (Al‐Ghafli et al., 2009; Evtimovska et al., 2009; Kleis et al., 2010; Kobayashi et al., 2014). Most patients who receive implant‐supported overdentures are elderly, and the average age of our participants in this survey was 72 years. Elderly patients, especially when fully edentate, often lack regular checkup visits because in their opinion, having no teeth means not requiring a dental checkup (Rentsch‐Kollar, Huber, & Mericske‐Stern, 2010). The loss of retention of the LOCATOR® attachment may actually be a blessing in disguise for the elderly patients as this helps bring the patient into the dental practice on a regular basis for recalls.

Maintenance was also related to the denture cap where the problems predominantly concentrated within the first year of denture insertion. The most frequent denture cap‐related problem was the need for repositioning of the denture cap. This problem seems to be a consequence of lab/intraoral processing. Attention needs to be exercised during the impression/intraoral processing procedures so as to avoid registering incorrect vertical positions of the attachments.

However, in no case, this study recorded a dislodgment of the denture cap from the prosthesis. This is surprising because the mechanical retention of the denture caps seems minimal but have been proven successful and sufficient in a long‐term clinical context, as demonstrated by this report.

### 4.4 Attachment wear higher in the maxilla than in the mandible

Wear of the LOCATOR® attachments was more pronounced in the maxilla than in the mandible. The reasons for this difference may be related to the anatomical shape of the alveolar ridges. The mandibular alveolar ridge allows almost always the insertion of parallel implants with an overdenture that can be inserted vertically and rarely the denture base of a mandibular overdenture that engages undercuts. Hence, the pull‐off forces for these overdentures are likely to be strictly vertical. In contrast, the maxillary alveolar ridge has often a small base, which implies that implants are more likely to present axial divergences (Al‐Ghafli et al., 2009; Martinez‐Lage‐Azorin, Segura‐Andres, Faus‐Lopez, & Agustin‐Panadero, 2013). Although the LOCATOR® attachment can compensate up to 40° of axial divergences, retention loss seems to be aggravated when these divergences are present (Srinivasan et al., 2016).

The more pronounced vestibular and mesial wear on the LOCATOR® attachment may be related to the insertion‐removal process of the maxillary IOD. The most common path of insertion and removal would be to first disengage the IOD in the posterior part and then pull on the front teeth. This would, however, result in a rather distal wear of the attachment. Hence, it can be speculated that patients tend to remove their dentures by either pulling on the front teeth or under the vestibular flanges, which could explain the observed wear pattern on the maxillary denture LOCATOR® attachments. However, this explanation remains speculative and needs to be clinically verified.

### 4.5 Attachment wear higher in maxillary IODs than all other prostheses

The results evinced that wear was higher in maxillary IODs than in ISRPDPs. This finding may be related to the denture kinetics as complete dentures have a higher mobility and longer lever arms for forces during chewing, but also during insertion and removal of the prosthesis. Whereas in ISRPDPs, the abutment teeth define a clear and unambiguous path of insertion; the angulations during insertion and removal therefore do not occur in the ISRPDPs as observed with IODs. In the maxilla, a minimum of four implants is recommended to retain an IOD because less than number is considered risky due to the maxillary bone quality (Weng & Richter, 2007). Therefore, the IOD kinetics favor implant wear due to lever forces and further explained by the increased number of implants required to sustain it.

### 4.6 Mostly mesial and vestibular AW for ISRDPs on >3 implants and mostly lingual AW ISRDPs on <3 implants

AW was the most prominent under prostheses engaged on >3 implants, when compared with implant prostheses using one or two implants. Although it could be assumed that IODs on two implants have larger lever forces acting on the implants and may show more wear, this was not confirmed by this study. It may, therefore, be assumed that the insertion and removal of the denture and the axial divergences of the implants account for the wear, rather than the denture kinetics during function.

The findings of this study demonstrate that the number of MCEs is related only to the time in use. The AW was found to be significant and related to type of prosthesis, jaw rehabilitated, and number of implants supporting the prosthesis. Therefore, both our primary and secondary hypotheses can only be partially rejected.

## 5 CONCLUSIONS

The most frequent MCE encountered with the LOCATOR® attachment was the loss of retention over time. Attachment wear was influenced by the jaw rehabilitated, whereas the wear sites were influenced by the number of implants supporting the prosthesis. Therefore, a modification in the attachment design along with an amelioration of the attachment surface may help decrease the maintenance needs and further enhance its clinical performance.

## FUNDING

The study was funded entirely by divisional funds allocated to the Division of Gerodontology and Removable Prosthodontics, University Clinics of Dental Medicine, University of Geneva, Switzerland for the planning, execution, or termination of the study. No other source of external funding was received.

## CONFLICTS OF INTERESTS

The authors wish to report that they have no conflicts of interests related to this study.

## STATEMENT OF AUTHORSHIP

All authors contributed to the four categories.

## APPENDIX A

**Table A1:** Mechanical complication events (MCEs) corrected for type of prosthesis

Groups	Number of participants (*n*)	Number of sites (implants survived; *n*)	Follow‐up period in months (Mean ± *SD*)	Number of mechanical complication events (N), number of patients (P) and share of patients (%) in subgroup experiencing at least one of those events														
				Attachment replacement		Attachment loosening		Denture cap‐related event		Loss of retention		Loss of insert		Implant fracture		Any event		Mean events per patient ± *SD*
				N	P (%)	N	P (%)	N	P (%)	N	P (%)	N	P (%)	N	P (%)	N	P (%)	
Ci/‐	12	46	58.2 ± 22.8	0	0 (0.0)	0	0 (0.0)	4	2 (16.7)	18	8 (66.7)	0	0 (0.0)	1	1 (8.3)	23	9 (75.0)	1.9 ± 1.7
‐/Ci	27	55	54.8 ± 22.8	3	3 (11.1)	0	0 (0.0)	4	4 (14.8)	47	18 (66.7)	1	1 (3.7)	0	0 (0.0)	55	19 (70.4)	2.0 ± 2.1
Pi/‐ or ‐/Pi	8	11	49.5 ± 19.8	0	0 (0.0)	1	1 (12.5)	0	0 (0.0)	10	6 (75.0)	0	0 (0.0)	0	0 (0.0)	11	6 (75.0)	1.4 ± 1.6
Total	47^a^	112	54.8 ± 22.1	3	3 (6.4)	1	1 (2.1)	8	6 (12.8)	75	32 (68.1)	1	1 (2.1)	1	1 (2.1)	89	34 (72.3)	1.9 ± 1.9
Mean ± *SD*				0.1 ± 0.2		0.0 ± 0.1		0.2 ± 0.5		1.6 ± 1.6		0.0 ± 0.1		0.0 ± 0.1		1.9 ± 1.9		
*p* value			0.479^b^	0.74^a^		0.17^a^		0.699^a^		1^a^		1^a^		0.426^a^		1^a^		

The *p* value corresponds to a Fisher's test, with the null hypothesis being that the rate of patients experiencing each type of mechanical complication is equal across the type of ISRDP. The Kruskal–Wallis test compared the distribution of the follow‐up period between the groups.; Ci/: upper implant supported CRDP; /Ci: lower implant supported CRDP; Pi/: upper implant supported PRDP; /Pi: lower PRDP; *SD*: standard deviation; *n*: number.

a Fisher's test.

b Kruskal–Wallis test.

**Table A2:** Mechanical complication events (MCEs) corrected for jaw rehabilitated

Groups	Number of participants (*n*)	Number of sites (implants survived; *n*)	Follow‐up period in months (Mean ± *SD*)	Number of mechanical complication events (N), number of patients (P) and share of patients (%) in subgroup experiencing at least one of those events														
				Attachment replacement		Attachment loosening		Denture cap‐related event		Loss of retention		Loss of insert		Implant Fracture		Any event		Mean events per patient ± *SD*
				N	P (%)	N	P (%)	N	P (%)	N	P (%)	N	P (%)	N	P (%)	N	P (%)	
Maxilla	15	49	56.5 ± 22.2	0	0 (0.0)	1	1 (6.7)	4	2 (13.3)	25	11 (73.3)	0	0 (0.0)	1	1 (6.7)	31	12 (80.0)	2.1 ± 1.7
Mandible	32	63	54.0 ± 22.3	3	3 (9.4)	0	0 (0.0)	4	4 (12.5)	50	21 (65.6)	1	1 (3.1)	0	0 (0.0)	58	22 (68.8)	1.8 ± 2.0
Total	47	112	54.8 ± 22.1	3	3 (6.4)	1	1 (2.1)	8	6 (12.8)	75	32 (68.1)	1	1 (2.1)	1	1 (2.1)	89	34 (72.3)	1.9 ± 1.9
Mean ± *SD*				0.1 ± 0.2		0.0 ± 0.1		0.2 ± 0.5		1.6 ± 1.6		0.0 ± 0.1		0.0 ± 0.1		1.9 ± 1.9		
*p* value			0.545^b^	0.541^a^		0.319^a^		1^a^		0.742^a^		1^a^		0.319^a^		0.503^a^		

The *p* value corresponds to a Fisher's test, with the null hypothesis being that the rate of patients experiencing each type of mechanical complication is equal across the type of jaw rehabilitated with the ISRDP. The Kruskal–Wallis test compared the distribution of the follow‐up period between the groups.; *SD*: standard deviation; *n*: number.

a Fisher's test.

b Kruskal–Wallis test.

**Table A3:** Mechanical complication events (MCEs) corrected for implant number per patient

Number of implant/s per prosthesis (*n*)	Number of participants (*n*)	Follow‐up period in months (Mean ± *SD*)	Number of mechanical complication events (N), number of patients (P) and share of patients (%) in subgroup experiencing at least one of those events														
			Attachment replacement		Attachment loosening		Denture cap‐related event		Loss of retention		Loss of insert		Implant Fracture		Any event		Mean events per patient ± *SD*
			N	P (%)	N	P (%)	N	P (%)	N	P (%)	N	P (%)	N	P (%)	N	P (%)	
1	6	51.0 ± 16.0	0	0 (0.0)	1	1 (16.7)	0	0 (0.0)	9	5 (83.3)	0	0 (0.0)	0	0 (0.0)	10	5 (83.3)	1.7 ± 1.8
2	30	55.0 ± 22.8	3	3 (10.0)	0	0 (0.0)	4	4 (13.3)	52	21 (70.0)	1	1 (3.3)	0	0 (0.0)	60	22 (73.3)	2.0 ± 2.0
3	1	44.7	0	0 (0.0)	0	0 (0.0)	0	0 (0.0)	0	0 (0.0)	0	0 (0.0)	0	0 (0.0)	0	0 (0.0)	0.0 (NA)
4	7	50.9 ± 27.1	0	0 (0.0)	0	0 (0.0)	4	2 (28.6)	9	4 (57.1)	0	0 (0.0)	1	1 (14.3)	14	5 (71.4)	2.0 ± 1.8
5	1	61.3	0	0 (0.0)	0	0 (0.0)	0	0 (0.0)	0	0 (0.0)	0	0 (0.0)	0	0 (0.0)	0	0 (0.0)	0.0 (NA)
6	2	78.8 ± 7.5	0	0 (0.0)	0	0 (0.0)	0	0 (0.0)	5	2 (100.0)	0	0 (0.0)	0	0 (0.0)	5	2 (100.0)	2.5 ± 2.1
Total	47	54.8 ± 22.1	3	3 (6.4)	1	1 (2.1)	8	6 (12.8)	75	32 (68.1)	1	1 (2.1)	1	1 (2.1)	89	34 (72.3)	1.9 ± 1.9
Mean ± *SD*			0.1 ± 0.2		0.0 ± 0.1		0.2 ± 0.5		1.6 ± 1.6		0.0 ± 0.1		0.0 ± 0.1		1.9 ± 1.9		
*p* value		0.478^b^	1^a^		0.213^a^		0.665^a^		0.322^a^		1^a^		0.362^a^		0.358^a^		

The *p* value corresponds to a Fisher's test, with the null hypothesis being that the rate of patients experiencing each type of mechanical complication is equal across number of implants. The Kruskal–Wallis test compared the distribution of the follow‐up period between the groups.; *SD*: standard deviation; *n*: number.

a Fisher's test.

b Kruskal–Wallis test.

**Table A4:** Mechanical complication events (MCEs) corrected for implant axial inclination

Implant axial inclination in degrees	Number of participants (*n*)	Number of sites (implants survived; *n*)	Number of mechanical complication events (N), number of patients (P) and share of patients (%) in subgroup experiencing at least one of those events														
			Attachment replacement		Attachment loosening		Denture cap‐related event		Loss of retention		Loss of insert		Implant fracture		Any event		Mean events per patient ± *SD*
			N	P (%)	N	P (%)	N	P (%)	N	P (%)	N	P (%)	N	P (%)	N	P (%)	
0–10	32	74	2	2 (6.3)	0	0 (0.0)	6	4 (12.5)	46	22 (68.8)	1	1 (3.1)	1	1 (3.1)	56	23 (71.9)	1.8 ± 1.8
10–20	15	38	1	1 (6.7)	1	1 (6.7)	2	2 (13.3)	29	10 (66.7)	0	0 (0.0)	0	0 (0.0)	33	11 (73.3)	2.2 ± 2.2
Total	47	112	3	3 (6.4)	1	1 (2.1)	8	6 (12.8)	75	32 (68.1)	1	1 (2.1)	1	1 (2.1)	89	34 (72.3)	1.9 ± 1.9
Mean ± *SD*			0.1 ± 0.2		0.0 ± 0.1		0.2 ± 0.5		1.6 ± 1.6		0.0 ± 0.1		0.0 ± 0.1		1.9 ± 1.9		
*p* value			1^a^		0.319^a^		1^a^		1^a^		1^a^		1^a^		1^a^		

In this table, in order to be able to have exclusive patient groups, patients were grouped according to the highest inclination in their implants, thus reducing the analysis to the patient level. In addition to this, the events were not site‐specific. The *p* value corresponds to a Fisher's test, with the null hypothesis being that the rate of patients experiencing each type of mechanical complication is equal regardless of the implant inclination.: standard deviation; *n*: number.

a Fisher's test.

**Table A5:** Mechanical complication events (MCEs) corrected for chewing efficiency

Variance of Hue scores ranges	Number of participants (*n*)	Number of sites (implants survived; n)	Number of mechanical complication events (N), number of patients (P) and share of patients (%) in subgroup experiencing at least one of those events														
			Attachment replacement		Attachment loosening		Denture cap related event		Loss of retention		Loss of insert		Implant Fracture		Any event		Mean events per patient ± *SD*
			N	P (%)	N	P (%)	N	P (%)	N	P (%)	N	P (%)	N	P (%)	N	P (%)	
≥0.501	1	2	0	0 (0.0)	0	0 (0.0)	0	0 (0.0)	1	1 (100.0)	0	0 (0.0)	0	0 (0.0)	1	1 (100.0)	1 (NA)
0.401–0.500	10	21	0	0 (0.0)	0	0 (0.0)	1	1 (10.0)	17	6 (60.0)	0	0 (0.0)	0	0 (0.0)	18	6 (60.0)	1.8 ± 2.6
0.301–0.400	15	39	2	2 (13.3)	1	1 (6.7)	5	3 (20.0)	27	11 (73.3)	1	1 (6.7)	0	0 (0.0)	36	12 (80.0)	2.4 ± 1.9
0.201–0.300	16	32	1	1 (6.3)	0	0 (0.0)	2	2 (12.5)	27	11 (68.8)	0	0 (0.0)	1	1 (6.3)	31	12 (75.0)	1.9 ± 1.7
≤0.100–0.200	5	18	0	0 (0.0)	0	0 (0.0)	0	0 (0.0)	3	3 (60.0)	0	0 (0.0)	0	0 (0.0)	3	3 (60.0)	0.6 ± 0.5
Total	47	112	3	3 (6.4)	1	1 (2.1)	8	6 (12.8)	75	32 (68.1)	1	1 (2.1)	1	1 (2.1)	89	34 (72.3)	1.9 ± 1.9
Mean ± *SD*			0.1 ± 0.2		0.0 ± 0.1		0.2 ± 0.5		1.6 ± 1.6		0.0 ± 0.1		0.0 ± 0.1		1.9 ± 1.9		
*p* value			0.741^a^		0.66^a^		0.834^a^		0.907^a^		0.660^a^		1.000^a^		0.748^a^		

The *p* value corresponds to a Fisher's test, with the null hypothesis being that the rate of patients experiencing each type of mechanical complication is equal across ranges of Hue scores.; *SD*: standard deviation; *n*: number.

a Fisher's test.

**Table A6:** Mechanical complication events (MCEs) corrected for wear

Highest Wear (always highest value per site implant)	Number of participants (*n*)	Number of sites (implants survived; *n*)	Number of mechanical complication events (N), number of patients (P) and share of patients (%) in subgroup experiencing at least one of those events														
			Attachment replacement		Attachment loosening		Denture cap related event		Loss of retention		Loss of insert		Implant Fracture		Any event		Mean events per patient ± *SD*
			N	P (%)	N	P (%)	N	P (%)	N	P (%)	N	P (%)	N	P (%)	N	P (%)	
No wear	33	65	3	3 (14.3)	0	0 (0.0)	3	3 (14.3)	20	11 (52.4)	1	1 (4.8)	0	0 (0.0)	27	12 (57.1)	1.3 ± 1.7
Minimal	11	16	0	0 (0.0)	0	0 (0.0)	0	0 (0.0)	14	4 (80.0)	0	0 (0.0)	0	0 (0.0)	14	4 (80.0)	2.8 ± 1.9
Moderate	20	36	0	0 (0.0)	1	1 (6.3)	4	2 (12.5)	32	12 (75.0)	0	0 (0.0)	1	1 (6.3)	38	13 (81.3)	2.4 ± 2.2
Advanced	5	8	0	0 (0.0)	0	0 (0.0)	1	1 (20.0)	9	5 (100.0)	0	0 (0.0)	0	0 (0.0)	10	5 (100.0)	2.0 ± 1.2
Total	69	125	3	3 (6.4)	1	1 (2.1)	8	6 (12.8)	75	32 (68.1)	1	1 (2.1)	1	1 (2.1)	89	34 (72.3)	1.9 ± 1.9
Mean ± *SD*			0.1 ± 0.2		0.0 ± 0.1		0.2 ± 0.5		1.6 ± 1.6		0.0 ± 0.1		0.0 ± 0.1		1.9 ± 1.9		
*p* value			0.43^a^		0.553^a^		1^a^		0.167^a^		1^a^		0.553^a^		0.187^a^		

Wear could not be assessed in the two failed implants belonging to patient 18 (sites 25 and 21), and in one implant belonging to patient 36 (24). Each patient was attributed to one of the four wear groups according to their highest wear (of any type) across their implants. Since a single patient can have implants with several types of wear, the sum of number of participants is higher than the number of patients assessed in the study. The *p* value corresponds to a Fisher's test, with the null hypothesis being that the rate of patients experiencing each type of mechanical complication is equal across the groups. Minimal: visible scratches on the attachment; Moderate: TiN coating lost and grey metal visible; Advanced: severe wear damaging the shape of the attachments; *SD*: standard deviation; *n*: number.

a Fisher's test.

**Table A7:** Attachment wear (AW) corrected for attachment height

Attachment height (mm)	Number of participants with at least one implant with attachment height (*n*)	Number of implants survived among those patients (*n*)	Number of sites (S) and share of sites (%) showing wear across the attachment split into four locations and overall (for any type of wear) per attachment. Mean wear ( $\bar{x}$) ± *SD*								
			Vestibular		Mesial		Distal		Lingual		Overall
			S (%)	$\bar{x}\;$ ± *SD*	S (%)	$\bar{x}\;$ ± *SD*	S (%)	$\bar{x}\;$ ± *SD*	S (%)	$\bar{x}\;$ ± *SD*	S (%)
1	21	35	9 (25.7)	0.46 ± 0.85	9 (25.7)	0.46 ± 0.85	5 (14.3)	0.46 ± 0.85	5 (14.3)	0.46 ± 0.85	13 (37.1)
2	24	34	12 (35.3)	0.59 ± 0.86	5 (14.7)	0.59 ± 0.86	8 (23.5)	0.59 ± 0.86	7 (20.6)	0.59 ± 0.86	17 (50.0)
3	17	24	11 (45.8)	0.79 ± 1.02	8 (33.3)	0.79 ± 1.02	6 (25.0)	0.79 ± 1.02	1 (4.2)	0.79 ± 1.02	12 (50.0)
4	8	14	8 (57.1)	1.21 ± 1.12	7 (50.0)	1.21 ± 1.12	2 (14.3)	1.21 ± 1.12	2 (14.3)	1.21 ± 1.12	11 (78.6)
5	3	3	3 (100.0)	2.00 ± 0.00	1 (33.3)	2.00 ± 0.00	1 (33.3)	2.00 ± 0.00	1 (33.3)	2.00 ± 000	3 (100.0)
6	1	1	0 (0.0)	0.00 ± 0.00	0 (0.0)	0.00 ± 0.00	0 (0.0)	0.00 ± 0.00	0 (0.0)	0.00 ± 0.00	0 (0.0)
Total	74	111	43 (38.7)	0.70 ± 0.96	30 (27.0)	0.43 ± 0.76	22 (19.8)	0.41 ± 0.86	16 (14.4)	0.31 ± 0.80	56 (50.5)
Number of worn implants per patient (Mean ± *SD*)			0.9 ± 1.2		0.6 ± 1		0.5 ± 0.8		0.3 ± 0.7		1.2 ± 1.3
*p* value			0.050^a^		0.151^a^		0.719^a^		0.399^a^		0.041^a^

The implant 24 belonging to patient 36 could not be evaluated for wear, bringing the total number of implants to 111 down from 112. The p‐value corresponds to a Fisher's test comparing the frequency of sites with wear among the different types of implants (according to attachment height), with the null hypothesis being that the share of sites with wear is equal across groups.; *SD*: standard deviation; *n*: number.

a Fisher's test.

**Table A8:** Attachment wear (AW) corrected for chewing efficiency

Variance of Hue scores ranges	Number of participants (*n*)	Number of sites (implants survived; *n*)	Number of patients (P) and share of patients (%) showing wear across the attachment split into four locations and overall (for any type of wear) per attachment. Mean wear ( $\bar{x}$) ± *SD*								
			Vestibular		Mesial		Distal		Lingual		Overall
			P (%)	$\bar{x}\;$ ± *SD*	P (%)	$\bar{x}\;$ ± *SD*	P (%)	$\bar{x}\;$ ± *SD*	P (%)	$\bar{x}\;$ ± *SD*	P (%)
≥0.501	1	2	0 (0.0)	0.00 ± 0.00	1 (100.0)	0.50 ± 0.00	1 (100.0)	0.50 ± 0.00	1 (100.0)	2.00 ± 0.00	1 (100.0)
0.401–0.500	10	21	3 (30.0)	0.28 ± 0.45	2 (20.0)	0.18 ± 0.37	1 (10.0)	0.30 ± 0.95	1 (10.0)	0.30 ± 0.95	3 (30.0)
0.301–0.400	15	39	9 (60.0)	0.91 ± 1.00	9 (60.0)	0.66 ± 0.70	6 (40.0)	0.59 ± 0.84	3 (20.0)	0.38 ± 0.90	6 (40.0)
0.201–0.300	16	31	8 (50.0)	0.63 ± 0.74	4 (25.0)	0.27 ± 0.60	5 (31.3)	0.49 ± 0.78	4 (25.0)	0.44 ± 0.79	7 (43.8)
≤0.100–0.200	5	18	3 (60.0)	0.85 ± 0.86	2 (40.0)	0.42 ± 0.69	1 (20.0)	0.16 ± 0.36	1 (20.0)	0.10 ± 0.22	2 (40.0)
Total	47	111	23 (48.9)	0.65 ± 0.80	18 (38.3)	0.39 ± 0.61	14 (29.8)	0.45 ± 0.79	10 (21.3)	0.39 ± 0.84	26 (55.3)
*p* value			0.542^a^		0.109^a^		0.289^a^		0.454^a^		0.636^a^

The *p* value corresponds to a Fisher's test comparing the frequency of patients with wear among the different categories of patients, with the null hypothesis being that the share of patients with wear is equal across groups.; *SD*: standard deviation; *n*: number.

a Fisher's test.
